# NMDA Receptor Mediates the Anticonvulsant Effect of Hydroalcoholic Extract of *Artemisia persica* in PTZ-Induced Seizure in Mice

**DOI:** 10.1155/2021/6422451

**Published:** 2021-08-04

**Authors:** Shakiba Nasiri-Boroujeni, Mohammad Rahimi-Madiseh, Zahra Lorigooini, Khadijeh Piroti, Mahmoud Rafieian-Koupaei, Hossein Amini-Khoei

**Affiliations:** Medical Plants Research Center, Basic Health Sciences Institute, Shahrekord University of Medical Sciences, Shahrekord, Iran

## Abstract

It is necessary to seek more effective sources to design new drug against epilepsy. This study aimed to evaluate the effect of hydroalcoholic extract of *Artemisia persica* on pentylenetetrazole- (PTZ-) induced seizure in male mice by investigating the possible role of the NMDA receptor and antioxidative stress effect. The phenolic profile of *A. persica* extract was determined by HPLC-DAD analysis. Mice were treated with normal saline or *A. persica* extract or pentobarbital or a subeffective dose of extract plus ketamine (NMDA receptor antagonist) and/or effective dose of extract plus NMDA. PTZ (90 mg/kg) was injected intravenously for induction of seizure. The seizure threshold was measured. Then mice were euthanized and the antioxidant capacity and the level of malondialdehyde (MDA) of the prefrontal cortex and serum were measured. The gene expression of NMDA receptor subunits (Nr2a and Nr2b) was determined by real-time PCR. Findings showed that *A. persica* extract increased the seizure threshold, increased antioxidant capacity, and decreased MDA levels in the serum and brain samples. *A. persica* extract reduced the expression of NMDA receptor subunits. The result showed that ketamine potentiated the effect of the subeffective dose of extract. HPLC analysis showed that quercetin had the highest flavonoid content and also caffeic acid had the highest content of the phenolic acids. *A. persica* extract probably via NMDA receptor exerts anticonvulsant properties.

## 1. Introduction

Epilepsy is one of the most common chronic neurological disorders that affect a significant percentage of the world's population [1]. Irregular and simultaneous discharges of neurons in the brain lead to seizure. Seizure is associated with a variety of symptoms, such as sudden loss of consciousness, muscle contraction, or sensory and behavioral changes [2]. Many epileptic patients do not respond well to antiepileptic drugs [3]. Common antiepileptic drugs cause various side effects [3, 4]. Thus, seeking rich sources for designing new drug with higher efficacy and lower side effects is a pressing need. Pentylenetetrazole (PTZ) is a selective chloride channel blocker coupled to the GABA-A receptor complex, commonly used as seizure inducer in rodents to evaluate antiepileptic agents [5]. It has been shown that PTZ increases intracellular calcium ion concentrations which are associated with N-methyl-d-aspartate (NMDA) receptors activation [6]. Previous studies showed that elevated calcium concentration in neurons prevents the inhibitory effects of GABA, which consequently led to neuronal stimulations and seizure [7].

In recent years, NMDA receptors have gained distinctive attention in the aspect of assessment of antiepileptic agents [8]. The role of NMDA receptors in the pathophysiology of seizure disorders has been determined previously [9]. The excitotoxicity of glutamatergic neurotransmission on the brain is mediated through the activation of NMDA receptors [10]. In this regard, it has been well known that NMDA receptor antagonists exert anticonvulsant effects [11]. Previous studies have shown that activation of NMDA receptors induces oxidative stress in neurons [12–14]. In this regard, it has been determined that blockade of NMDA receptors prevents the oxidative stress biomarkers [15]. Zhu et al. showed that Nr2b subunit of NMDA receptor mediates oxidative stress in the hippocampal neurons in PTZ-kindling-induced hippocampal oxidative stress [16]. Ample evidence demonstrated that mitochondrial dysfunction and oxidative stress play pivotal role in the pathophysiology of seizures. In this concept, research studies determined that antioxidants exerted anticonvulsant properties [17–19].

Recently, the use of medicinal plants in the treatment of various diseases including seizure is increasing [20]. There are more than 250 species of *Artemisia* in the world, of which about 34 species grow in Iran [21, 22]. *A. persica* is one of the valuable medicinal plants with the local name of Persian Joshan [23, 24]. *A. persica* belongs to the Anthemideae tribe and the Asteraceae family [24]. Dried flowers and aerial part of *A. persica*, as well as its compounds including essential oils, minerals, resin, santonin, and artemisinin, are famous in traditional medicine [23, 25]. The roots, stems, and leaves of *A. persica* are used to treat coughs, fevers, loss of appetite, colic, headaches, earaches, and parasitic diseases and malaria [26]. Previous studies have determined that ethanolic extract of *A. persica* possessed neuroprotective, antidepressant-like, and anxiolytic-like effects, as well as improving cognition and memory in various experimental models in rodents [27–30]. The antiepileptic effect of extract of *A. persica* has been reported previously; however, the underlying mechanisms involved in this effect are not determined [28].

In this study, considering (1) the incomplete effects of antiepileptic drugs in some patients as well as their side effects, (2) the involvement of NMDA receptors in the pathophysiology of seizures, and (3) the reported pharmacological effects for *A. persica*, we intend to investigate the anticonvulsant effect of hydroalcoholic extract of *A. persica* in PTZ-induced seizures in mice by considering the possible role of NMDA receptors.

## 2. Material and Methods

### 2.1. Plant Material and Preparation of Hydroalcoholic Extract

*A. persica* was collected from Piazi mountain heights, Shir Mard village in Falard Rural District, Lordegan County, Chaharmahal and Bakhtiari Province, Iran. It was identified by an expert botanist (Shirmardi, Hamzeh Ali, PhD., Research Center of Agriculture and Natural Resources, P.O. Box 415, Shahrekord, Iran, and Shahrokhi, Asghar; Education Organization, Chaharmahal and Bakhtiari Province, Shahrekord, Iran). A reference sample was kept in the Herbarium of Medicinal Plants Research Center of Shahrekord University of Medical Science with voucher herbarium (specimen no. 1008). Extraction was performed by the maceration method. The powdered sample of *A. persica* was mixed with 70% alcohol in a ratio of 1 : 5, after 72 hours, the extract was filtered and concentrated by a rotary evaporator apparatus. Finally, it was dried in an incubator at 37°C [31].

### 2.2. HPLC Analysis of Flavonoids and Phenolic Acids

The analysis of flavonoids and phenolic acids was performed by HPLC method according to the method of Jamshidi-kia et al. (2020). The qualitative analysis was performed using reverse phase high performance liquid chromatography (RP-HPLC) coupled with UV-visible detector (UV PDA 2800) and column C18 (5 *µ*m particle size, i.d. 250 mm × 4.6 mm). First, after dissolving 2.5 mg of dry extract in 1 ml methanol and water in a ratio of 1 : 4 to prepare a concentration of 2500 ppm and the injection volume was 20 *µ*L, the samples were injected into HPLC and isolated by the fixed phase C18 and the binary mobile phase of deionized water and methanol containing trifluoroacetic acid 0.05%. Detection was performed by scanning from 190 to 800 nm and reading in the range of 280–372 nm. The results were calculated using the calibration curve equation of analytical standards of caffeic acid, rutin, naringin, apigenin, luteolin, and quercetin to identify and determine the amount. The results were expressed as µg/mg DW of extract [32].

### 2.3. Study Design

In this experimental study, 56 male NMRI mice with the weight range of 25–35 g and approximately 6–8 weeks of age were divided into 7 groups (*n* = 10) as follows.

Group 1 received normal saline at a dose of 1 ml/kg, groups 2–4 received *A. persica* ethanolic extract at doses of 100, 200, and 400 mg/kg, i.p. [28], and group 5 received subeffective dose of *A. persica* ethanolic extract plus subeffective dose of ketamine (NMDA receptor antagonist, 0.5 mg/kg) [33]. The sixth group received the effective dose of *A. persica* extract plus the effective dose of NMDA agonist (150 mg/kg) [34]. The last group received phenobarbital (as a standard anticonvulsant agent) at a dose of 4 mg/kg [35]. All agents were administrated intraperitoneally (i.p.) one hour before PTZ induces the seizure. Dose and time of drug administrations were chosen based on our pilot study as well as previous studies [33–36].

### 2.4. Determination of Seizure Threshold

To induce seizure, a 30-gauge butterfly needle was inserted and fixed to the tail vein of mice. Mice were allowed to move freely. PTZ solution (0.5%) was slowly infused into the tail vein at a constant rate of 1 ml/min using seizure pump (NE 1000, New Era Pump System, Inc.). The injection was stopped as soon as the anterior limb clonus was seen. The minimum dose of PTZ (mg/kg of mice weight) needed for seizure induction was considered as an index of clonic seizure threshold. In this method, the seizure threshold is PTZ-dose-dependent and time-related [37].

### 2.5. Determination of Total Antioxidant Level of Brain and Serum

The FRAP (ferric reducing ability of plasma) in prefrontal cortex and serum samples was determined at 37°C and pH 3.6 according to the previously described method [38]. In brief, absorbance was measured after 30 min and reported as a proportion to the combined ferric reducing/antioxidant power of the antioxidants in protein, and the results were expressed as mmol Fe_2+_/mg protein.

### 2.6. Measurement of Malondialdehyde (MDA) Level of Prefrontal Cortex  and  Plasma

MDA was measured in the serum and prefrontal cortex samples based on the previously described method [39]. In brief, using thiobarbituric acid (TBA) method based on the development of colorful chromophores following the reaction of TBA with MDA, the concentration of MDA was measured. The absorbance of the supernatants was measured at 562 nm for prefrontal cortex samples and at 532 nm for serum samples using an ELISA reader. The MDA concentration was reported as *μ*mol/L (for MDA formed per litres of serum) and nmol/mg protein (for MDA formed per mg protein of the prefrontal cortex).

### 2.7. Gene Expression in RT-PCR

At the end of the experiment, the mice were sacrificed under deep anaesthesia using diethyl ether, and the prefrontal cortex was removed and the gene expression of subunits of NMDA receptor (Nr2a and Nr2b) was assessed by real-time PCR. The reaction for each gene was tripled and repeated twice. The required specific primers were designed using Primer 3 software version 0.4.0 (Table 1), the H2afz gene was considered as normalizer, and the rate of change in expression of the desired genes was compared with the control group. Finally, the data were calculated using the PFAFFL formula [40].

### 2.8. Statistical Analysis

Statistical analysis was performed using PRISM software and the results were presented as mean ± SEM. One-way ANOVA and Tukey's post hoc tests were used for analysis. *P* values < 0.05 were considered to be statistically significant.

## 3. Results

### 3.1. HPLC Analysis

Flavonoids and phenolic acids were quantified by the HPLC method and compounds (caffeic acid, rutin, naringin, apigenin, luteolin, and quercetin) were identified and quantified as µg per 0.02 g of dried extract. The results are shown in Table 2. The flavonoid content of the extract was free of rutin and naringin. Also, the highest flavonoid content is related to quercetin (17.3 ± 0.01 *μ*g/0.02 g of dry extract). Caffeic acid was 46.5 ± 0.2 *μ*g/0.02 g of dried extract and had the highest content of phenolic acids.

### 3.2. Evaluation of Seizure  Tolerance

**Figure 1:**
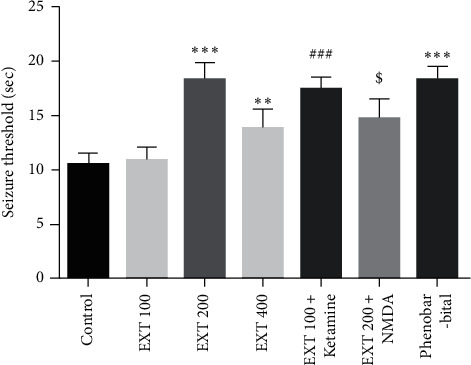
The seizure threshold in the experimental groups. Values are presented as the mean ± SEM from 8 animals and were analyzed by one-way ANOVA followed by Tukey's post hoc test. ^∗∗^*P* < 0.01 and ^∗∗∗^*P* < 0.001 compared to the control group; ^###^*P* < 0.001 compared with the group received 100 mg/kg of *A. persica* extract and ^$^*P* < 0.05 compared with the group received 200 mg/kg of *A. persica* extract. EXT : *A. persica* extract.

Figure 1 shows the onset time of seizures (seizure threshold) in the experimental groups. The seizure threshold significantly increased in the groups that received *A. persica* extract at the doses of 200 and 400 mg/kg in comparison to the control group (*P* < 0.001 and *P* < 0.01, respectively). Also, the seizure threshold significantly increased in the group that received the subeffective dose of *A. persica* extract (100 mg/kg) plus ketamine (as NMDA antagonist) compared to the group that received the subeffective dose of *A. persica* extract alone (*P* < 0.001). The seizures threshold significantly decreased in the group that received an effective dose of *A. persica* extract (200 mg/kg) plus NMDA (as NMDA agonist) compared to the group that received an effective dose of *A. persica* extract alone (*P* < 0.05). In addition, administration of phenobarbital significantly increased the seizure threshold in comparison with the control group (*P* < 0.001).

### 3.3. Serum Antioxidant Capacity

**Figure 2:**
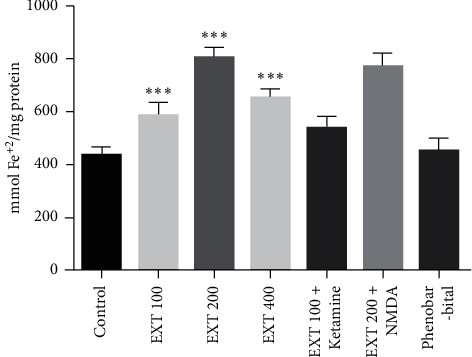
Serum antioxidant capacity in the experimental groups. Values are presented as the mean ± SEM from 8 animals and were analyzed by one-way ANOVA followed by Tukey's post hoc test. ^∗∗∗^*P* < 0.001 compared to the control group. EXT: *A. persica* extract.

According to the results (Figure 2 ), the antioxidant capacity of serum in the groups received *A. persica* extract at the doses of 100, 200, and 400 mg/kg significantly increased as compared to the control group (*P* < 0.001). However, the antioxidant capacity of serum in the groups that received *A. persica* extract plus NMDA receptor agonist/antagonists was not significantly different from their counterparts.

### 3.4. Prefrontal Cortex Antioxidant Capacity

**Figure 3:**
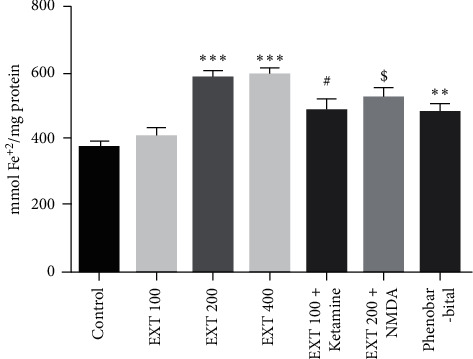
Prefrontal cortex antioxidant capacity in the experimental groups. Values are presented as the mean ± SEM from 8 animals and were analyzed by one-way ANOVA followed by Tukey's post hoc test. ^∗∗^*P* < 0.01 and ^∗∗∗^*P* < 0.001 compared to the control group; ^#^*P* < 0.05 compared with the group received 100 mg/kg of *A. persica* extract; ^$^*P* < 0.05 compared with the group received 200 mg/kg of *A. persica* extract. EXT: *A. persica* extract.

As shown in Figure 3 , the antioxidant capacity of the prefrontal cortex in the groups that received *A. persica* extract at doses of 200 and 400 mg/kg significantly increased compared to the control group (*P* < 0.001). The antioxidant capacity of the prefrontal cortex in the group that received effective dose of *A. persica* extract (200 mg/kg) plus NMDA receptor agonist significantly decreased compared to the group that received effective dose of *A. persica* extract alone (*P* < 0.05). The antioxidant capacity of the prefrontal cortex in the group that received subeffective dose of *A. persica* extract (100 mg/kg) plus ketamine significantly increased as compared to the group received subeffective dose of *A. persica* extract alone (*P* < 0.05). In addition, administration of phenobarbital significantly increased antioxidant capacity of the prefrontal cortex compared with the control group (*P* < 0.01).

### 3.5. Serum MDA Levels

**Figure 4:**
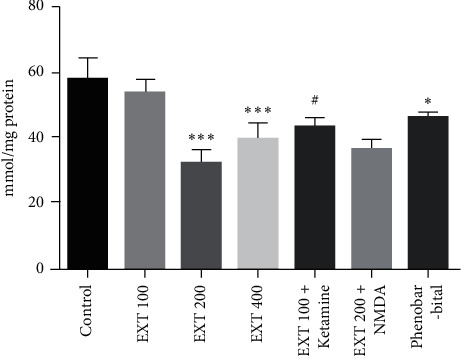
MDA levels of serum samples in the experimental groups. Values are presented as the mean ± SEM from 8 animals and were analyzed by one-way ANOVA followed by Tukey's post hoc test. ^*∗*^*P* < 0.05 and ^∗∗∗^*P* < 0.001 indicate significant differences with the control group; ^#^*P* < 0.05 compared with the group received *A. persica* extract at the dose of 100 mg/kg. EXT: *A. persica* extract.

As Figure 4 shows, serum MDA levels in the groups that received *A. persica* extract at doses of 200 and 400 mg/kg significantly decreased compared to the control group (*P* < 0.001). MDA level in serum samples of the group that received ketamine plus the subeffective dose of *A. persica* extract significantly decreased as compared to the group that received the subeffective dose of *A. persica* extract alone (*P* < 0.05). Furthermore, treatment with phenobarbital significantly decreased compared to the control group (*P* < 0.05).

### 3.6. Prefrontal Cortex MDA Levels

**Figure 5:**
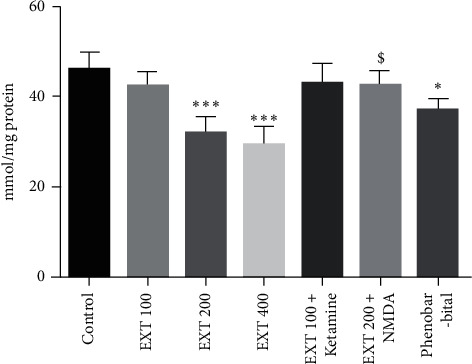
MDA levels of the prefrontal cortex samples in the experimental groups. Values are presented as the mean ± SEM from 8 animals and were analyzed by one-way ANOVA followed by Tukey's post hoc test. ^*∗*^*P* < 0.0 and ^∗∗∗^*P* < 0.001 indicate significant differences with the control group; ^$^*P* < 0.05 compared with the group that received *A. persica* extract at the dose of 200 mg/kg. EXT: *A. persica* extract.

According to Figure 5 , the prefrontal cortex MDA levels in the groups that received *A. persica* extract at doses of 200 and 400 mg/kg significantly decreased in comparison with the control group (*P* < 0.001). MDA level in the prefrontal cortex samples of the group that received NMDA plus the effective dose of *A. persica* extract significantly increased compared to the group received the effective dose of *A. persica* extract alone (*P* < 0.05). Furthermore, treatment with phenobarbital significantly decreased compared to the control group (*P* < 0.05).

### 3.7. Gene Expression of Nr2a and Nr2b Receptors

**Figure 6:**
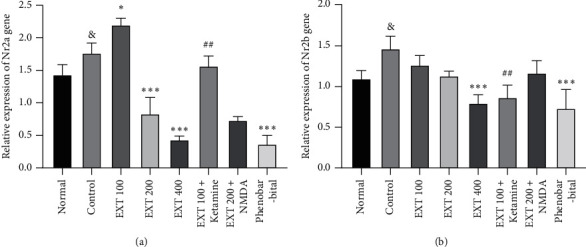
The expression rate of NMDA receptor. (a) Nr2a subunit and (b) Nr2b subunit genes in the experimental groups. ^&^*P* < 0.05 compared with the normal group. ^*∗*^*P* < 0.05 and ^∗∗∗^*P* < 0.001 compared with the control group; ^##^*P* < 0.01 compared with group received extract at dose of 100 mg/kg. EXT: *A. persica* extract.

As shown in Figure 6(a) , gene expression of the Nr2a subunit of NMDA receptor significantly increased in the group received PTZ (control group) as compared to the normal group (group that received normal saline only) (*P* < 0.05). The expression of the Nr2a gene in the group that received 100 mg/kg of *A. persica* extract significantly increased compared to the control group (*P* < 0.05). Also, the expression of Nr2a in the groups that received extract at doses of 200 and 400 mg/kg, as well as the phenobarbital, significantly reduced compared to the control group (*P* < 0.001). In the group that received the subeffective dose of the extract (100 mg/kg) plus ketamine, the expression of Nr2a significantly reduced compared to the group that received the subeffective dose of extract alone (*P* < 0.01).

According to Figure 6(b), the gene expression of the Nr2b subunit of NMDA receptor significantly increased in the group received PTZ (control group) compared to the normal group (group that received normal saline only) (*P* < 0.05). Also, the expression of Nr2b in the groups that received extract at the dose of 400 mg/kg as well as the phenobarbital significantly reduced in comparison to the control group (*P* < 0.001). In the group that received the subeffective dose of the extract (100 mg/kg) plus ketamine, the expression of Nr2b significantly reduced compared to the group that received the subeffective dose of extract alone (*P* < 0.01).

## 4. Discussion

The present study aimed to evaluate the effect of hydroalcoholic extract of *A. persica* in pentylenetetrazole-induced seizures considering the possible involvement of NMDA receptors in male mice. Results showed that *A. persica* extract at doses of 200 and 400 mg/kg significantly increased the seizure threshold. Also, coadministration of NMDA antagonist (ketamine) with the subeffective dose of the extract (100 mg/kg) potentiated the anticonvulsant effect of the subeffective dose of extract. In addition, coinjection of NMDA agonist with the effective dose of the extract (200 mg/kg) mitigated the anticonvulsant effect of the effective dose of extract. We found that *A. persica* extract significantly increased serum and prefrontal cortex antioxidant capacity and significantly decreased serum and prefrontal cortex MDA levels. Furthermore, gene expression of Nr2a and Nr2b significantly increased in the PTZ group while administration of *A. persica* extract significantly decreased the expression of these genes.

Previous studies have demonstrated that quercetin through its neuroprotective and antineuroinflammatory effects possessed anticonvulsant effects in seizure models in rodents [41, 42]. In the quantification of flavonoids and phenolic acids, the *A. persica* extract is free of rutin and naringin. However, quercetin had the highest flavonoid content and caffeic acid had the highest content of the phenolic acids.

In a study conducted by Keshavarzian et al. on the anticonvulsant effects of *Artemisia aucheri*, they obtained similar results in line with our finding. They concluded that the hydroalcoholic extract of *Artemisia aucheri* increased seizure threshold in PTZ-induced seizures in a dose- and time-dependent manner [43].

A 2004 study by Kordjazy et al., which is in line with our results, found that PTZ increased intracellular calcium ion concentrations as well as the expression of NMDA receptors [44]. Another study by Li et al. in 2016 found that the anticonvulsant effect of the 5-HT3 antagonist was at least partially inhibited by coadministration of the NMDA receptor agonists [45]. Finding of the present study determined that the gene expression of NMDA receptor subunits (Nr2a and Nr2b) increased following induction of seizure.

A study by Woo et al. concluded that *Artemisia capillaris* has an anticonvulsant effect that can be attributed to the enhancement of GABA activity [46]. A study by Khan et al. also showed that carnosol, ursolic acid, and oleanolic acid in *Artemisia indica* L,. by balancing the GABA-A receptors, induced antidepressant and anticonvulsant effects in mouse models [36].

Previous studies have demonstrated that an increase in seizure vulnerability is associated with alterations in NMDA regulation in the prefrontal cortex [47]. The literature determined that administration of NMDA receptor antagonists including ketamine and MK-801 exerted an anticonvulsant effect. In this regard, it has been shown that increased expression of NMDA receptor subunits in the prefrontal cortex accounts for increased seizure susceptibility in the PTZ model of the seizure [48, 49]. Furthermore, Frasca et al. have shown that dysregulation of NMDA receptors in the limbic area provokes excitatory state and epileptogenesis [50]. Our findings showed that administration of ketamine (NMDA receptor antagonist) potentiated the anticonvulsant effect of the subeffective dose of *A. persica*. Besides, coadministration of ketamine plus subeffective dose of *Artemisia persica* reduced the expression of NMDA receptor subunits (Nr2a and Nr2b) in the prefrontal cortex. This outcome showed that partially, at least, the anticonvulsant effect of *A. persica* is mediated through NMDA receptors.

It has been shown that NMDA receptors mediated an oxidative stress in neurons [51]. In this regard, it has been determined that NMDA receptor antagonists prevent the oxidative stress biomarkers [15]. Zhu et al. showed that Nr2b subunit of NMDA receptor mediates oxidative stress in the hippocampal neurons in PTZ-kindling-induced hippocampal oxidative stress [16]. Evidence has demonstrated that oxidative stress plays pivotal role in the pathophysiology of seizures. Researches have determined that antioxidants exerted anticonvulsant effects [17–19]. In line with aforementioned studies, our findings showed that *Artemisia persica* extract reduced the MDA levels and increased total antioxidant capacity in the serum and prefrontal cortex samples in PTZ-induced mice. We showed that NMDA receptors agonist and antagonists, respectively, attenuated and potentiated the antioxidative effects of *Artemisia persica* extract. Our results showed that antioxidant capacity in the serum could not be related to NDMA receptors. However, evaluation of prefrontal cortex antioxidant capacity showed that NMDA receptors mediated, partially at least, the effect of A. persica extract on antioxidant capacity in the prefrontal cortex. These findings indicated that, at least in part, the antioxidative effects of *Artemisia persica* extract were mediated via inactivation of NMDA receptors.

## 5. Conclusion

The findings of the present study showed that *A. persica* increases seizure tolerance in the PTZ model of seizure in mice. We found that at least the anticonvulsant effect of *A. persica* is mediated through NMDA receptors. Results determined that the hydroalcoholic extract of *A. persica* significantly increased the serum and prefrontal cortex antioxidant capacity and significantly reduced the amount of MDA in plasma and prefrontal cortex samples, indicating that antioxidant properties of *A. persica* may be involved in its anticonvulsant effect.

## Figures and Tables

**Table 1:** Primer sequences used in PCR amplification.

Primer	Forward sequence	Reverse sequence
H2afz	TCATCGACACCTGAAATCTAGGA	AGGGGTGATACGCTTTACCTTTA
Nr2*α*	CTCAGCATTGTCACCTTGGA	GCAGCACTTCTTCACATTCAT
Nr2b	CTACTGCTGGCTGCTGGTGA	GACTGGAGAATGGAGACGGCTA

**Table 2:** Flavonoid content of *A. persica* extract in *µ*g per 0.02 g of dry extract.

Naringin	Apigenin	Luteolin	Quercetin	Rutin	Caffeic acid
—	1.70 ± 0.05	11.1 ± 0.01	17.3 ± 0.01	—	46.5 ± 0.02

## Data Availability

Data regarding the present study are available at Medical Plants Research Center, Shahrekord University of Medical Sciences.
